# Basal ganglia—thalamus and the “crowning enigma”

**DOI:** 10.3389/fncir.2015.00071

**Published:** 2015-11-04

**Authors:** Marianela Garcia-Munoz, Gordon W. Arbuthnott

**Affiliations:** Okinawa Institute of Science and Technology Graduate UniversityOkinawa, Japan

**Keywords:** ventromedial, ventrolateral, ventral anterior, midline-intralaminar, basal ganglia, motor cortex

## Abstract

When Hubel (1982) referred to layer 1 of primary visual cortex as “… a ‘crowning mystery’ to keep area-17 physiologists busy for years to come …” he could have been talking about any cortical area. In the 80’s and 90’s there were no methods to examine this neuropile on the surface of the cortex: a tangled web of axons and dendrites from a variety of different places with unknown specificities and doubtful connections to the cortical output neurons some hundreds of microns below. Recently, three changes have made the crowning enigma less of an impossible mission: the clear presence of neurons in layer 1 (L1), the active conduction of voltage along apical dendrites and optogenetic methods that might allow us to look at one source of input at a time. For all of those reasons alone, it seems it is time to take seriously the function of L1. The functional properties of this layer will need to wait for more experiments but already L1 cells are GAD67 positive, i.e., inhibitory! They could reverse the sign of the thalamic glutamate (GLU) input for the entire cortex. It is at least possible that in the near future normal activity of individual sources of L1 could be detected using genetic tools. We are at the outset of important times in the exploration of thalamic functions and perhaps the solution to the crowning enigma is within sight. Our review looks forward to that solution from the solid basis of the anatomy of the basal ganglia output to motor thalamus. We will focus on L1, its afferents, intrinsic neurons and its influence on responses of pyramidal neurons in layers 2/3 and 5. Since L1 is present in the whole cortex we will provide a general overview considering evidence mainly from the somatosensory (S1) cortex before focusing on motor cortex.

## Layer 1: The Crowning Enigma

Our interest in layer 1 (L1) of cortex sprang from the knowledge that it was the final destination of basal ganglia output. In spite of the fact that the whole cortex is represented in striatum and movement is certainly not its only function, here we review thalamic output to motor cortex because of our long-standing interest in movement and the basal ganglia. Similarly L1 is not only present in motor cortex but invests the complete cortex. In theorizing about basal ganglia and their influence on the “crowning enigma” it is as important to remember that movement is only the most obvious output and the easiest outcome to measure from both the dark basements of the brain and its crowing glory. With its plastic spines and input from so many parts of the environment L1 might be the best place to provide the necessary context for movements, or for perceptions, both of which, like all decisions of the cortex, need information from many sources including historical experience that can be coded in the pattern of spines.

## Neurons Intrinsic to Layer 1

L1 is recognized throughout the entire cerebral cortex. Cajal (1890) described for the first time horizontal cells in L1 later called Cajal-Retzius and Lorente De Nó (1922) using Golgi staining in mouse auditory cortex provided the first evidence of “non-specific” thalamic afferents to L1. For a review see Marin-Padilla and Marin-Padilla (1982).

From work perform ed in mice and rat neocortex (rostral, central and caudal areas) three types of neurons in L1 are described as non-pyramidal GABAergic: Cajal-Retzius, elongated neurogliaform and single bouquet. Cajal-Retzius are the earliest born (embryonic 10–11 days; Soda et al., 2003; Anstotz et al., 2014) and they have an oval shape with a prominent long spiny dendrite (two occasionally) that runs horizontally along L1 (Imamoto et al., 1994). Their horizontal axon extends about 1.7 mm and serves as anchor of dendritic tufts of pyramidal neurons of layers 2/3 and 5 (Anstotz et al., 2014). These neurons participate in layering and connectivity during development (Zecevic and Rakic, 2001; Soda et al., 2003). During the second postnatal week most Cajal-Retzius cells suffer apoptotic death (del Río et al., 1995; Chowdhury et al., 2010) and have nearly disappeared at P14 (Anstotz et al., 2014).

The elongated neurogliaform type comprises 30–40% of neurons in L1, they have a characteristic dense axonal arbor confined to L1 and are coupled electrically. These cells express GABA_Aδ_ receptors and mostly display non-adapting late spiking action potentials. In the monkey sensory-motor cortex intense GABA_A_ immunostaining outlines somas of pyramidal and non-pyramidal cells in layers 1–3 (Huntley et al., 1990). Single bouquet cells express vasoactive intestinal peptide (VIP) and mostly display adapting early spiking to depolarizing current injections (Jiang et al., 2013; Ma et al., 2014).

Following the Petilla terminology for interneuron firing patterns (Ascoli et al., 2008) there are four different types of interneurons in L1: neurogliaform, classical-accomodating, fast-spiking and burst-spiking (Wozny and Williams, 2011). The most common type is the fast spiking, with no frequency adaptation and pronounced fast afterhyperpolarizations (Zhou and Hablitz, 1996; Wozny and Williams, 2011; Li et al., 2012; Muralidhar et al., 2013). Correlation of function and morphology with colocalization of neuronal markers and specific neuronal proteins has produced four different subtypes of agranular neocortical GABAergic neurons. Two are found in L1: the calretinin/alpha-actin-2 and somatostatin subtypes (Kubota et al., 2011).

## Afferents to Layer 1

It is estimated that 4000–5000 glutamate (GLU) containing axons reach any given square millimeter of rat L1 (Rubio-Garrido et al., 2009) to selectively target apical dendritic tufts (Herkenham, 1986; Arbuthnott et al., 1990; Lu and Lin, 1993), Figure 1 illustrates the extension of neocortical L1 projections from ventromedial (VM) that include forelimb and hindlimb areas. Dendritic tufts of layer 5 corticospinal, corticostriatal and corticothalamic neurons are all subject to modulation from L1 (Gao and Zheng, 2004). Moreover, important modulation of dendritic tufts of layer 2/3 pyramidal neurons takes place in L1 (see “Responses Mediated by Activation of Layer 1” Section).

**Figure 1:**
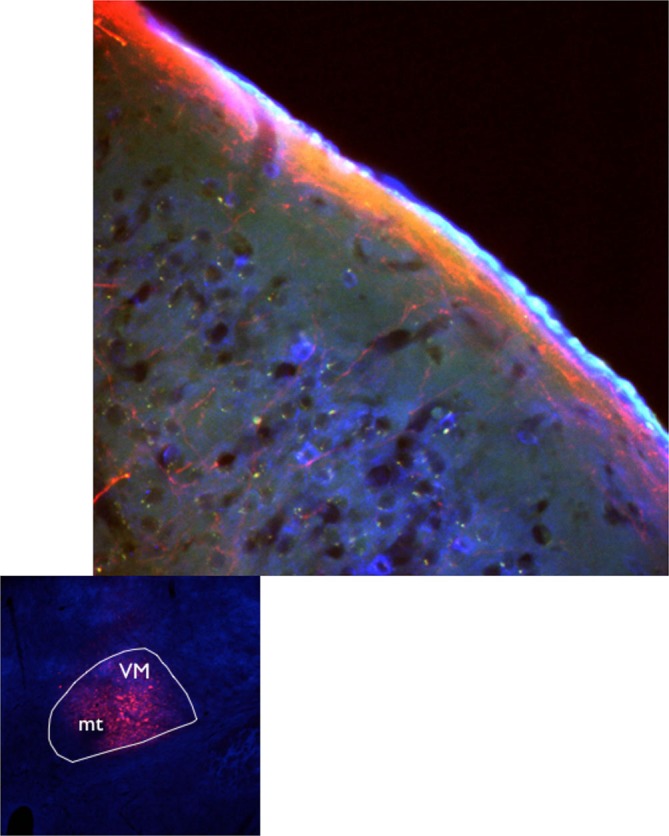
**Axons from the ventromedial nucleus of the motor thalamus delineate layer 1 in motor cortex.** Axon terminal fields in cortical L1 seen after VM injection of AAV10 containing turbo red and GCamp6 in C57Bl6J mice (Jáidar Benavides and Arbuthnott, 2013; Jáidar et al., 2015).

In motor cortex afferents reaching L1 are exclusively from motor thalamus (MT) and midline rhomboid nucleus (Ohtake and Yamada, 1989; Van Der Werf et al., 2002; Vertes et al., 2015). Other midline/intralaminar nuclei (i.e., centrolateral, centromedian, paracentral, posterior and parafascicular) terminate although not exclusively, in L1 of motor related areas (Royce and Mourey, 1985; Royce et al., 1989; Jones, 2007; Mohammed and Jain, 2014).

### Neuronal Processes that Mingle in Layer 1

Axons that run along L1 originate in higher cortical areas, thalamic specific and non-specific nuclei (Mitchell and Cauller, 2001) and brainstem specific neurotransmitter producing nuclei. Norepinephrine (NE) originates in the pontine locus coeruleus, serotonin (5HT) in the midbrain raphe nuclei, dopamine (DA) in the ventral mesencephalon and acetylcholine (ACh) in the basal forebrain. In general these neurotransmitter-containing fibers enter below layer 6 and ascend sending collateral branches at all levels. L1 is particularly filled with dense axonal terminals and long branching collaterals (Levitt and Moore, 1978, 1979). Cortical NE innervates the marginal zone at embryonic 18–21 days and its participation in pyramidal cell development and layering was highlighted following locus coeruleus lesions in newborn rats (Felten et al., 1982). Similarly, 5HT is related to neuronal development, differentiation and migration (Rubenstein, 1998). The participation of DA and ACh will be indicated below (see “Modulatory Role of Neurotrasmitters Released in L1” Section) associated to responses mediated by activation of L1.

Other neuronal processes in L1 come from cortical interneurons, mainly from axons of somatostatin-positive Martinotti cells contained in layers 2–6 (Thomson and Lamy, 2007; Muralidhar et al., 2013), vertical dendrites from bipolar interneurons (layers 1–3) that run horizontally once in L1, and apical dendrites of layer 5 pyramidal cells (Larsen and Callaway, 2006) and layer 2/3 (Walcott and Langdon, 2001). Intrinsic axonal arborizations from L1 run either in the horizontal plane along the layer or descend in the vertical plane to frequently synapse with interneurons of deeper layers (Zhu and Zhu, 2004; Jiang et al., 2013).

### Motor Thalamus

Motor thalamus (MT) is considered the area where afferents from globus pallidus (GPi or entopeduncular nucleus, EP), substantia nigra reticulata (SNR) and deep cerebellar nuclei form terminal fields in separate nuclei of the ventral thalamus: ventrolateral (VL), ventral anterior (VA) and ventromedial (VM). According to Scheibel and Scheibel (1967) the best way to conceptualize MT is to look at a horizontal section of the brain through the rostral half of thalamus.

#### MT Inputs

Terminal sites of afferent axons to MT are conserved across species (Antal et al., 2014) and establish multiple synapses with neurons in VM (Kultas-Ilinsky and Ilinsky, 1990; Kuroda and Price, 1991; Sakai et al., 1998; Tsumori et al., 2002; Bodor et al., 2008).

The use of a new anatomical technique with a resolution like “the old Golgi staining” (Furuta et al., 2001) has refined previous findings of inputs to MT (Deniau et al., 1978; Uno et al., 1978; Bava et al., 1979; MacLeod et al., 1980; Chevalier and Deniau, 1982; Matsuda and Nakamura, 1982; Ueki, 1983). As a result the VA/VL complex is divided in two sections: the rostromedial area immunoreactive to calbindin and GAD67 and the caudolateral area immunoreactive to VGluT2. These results sparked the idea of associating the neurotransmitter markers with the sites of origin calling the GABAergic GAD67-immunoreactive neurons the “inhibitory zone” and the glutamatergic VGluT2-immunoreactive neurons the “excitatory zone”. It is important to note that although immunureactivities to GAD67 and VGluT2 vary in intensity, they can be found at variable levels throughout MT. VM and VA/VL contain axon terminals of both GABA and GLU in different proportions. GABAergic terminals from SNR and GPi(EP) terminate in VM and the rostroventral VA/VL and cerebellar GLU terminals in the caudodorsal portion of VA/VL (Kuramoto et al., 2011).

In rats and monkeys, the GAD67-immunoreactive axon terminals are large (Bodor et al., 2008; Kuramoto et al., 2011) with a synaptic arrangement of the typical thalamic “detonator or driver”-type input that favors neurotransmitter spillover and volume transmission (Destexhe and Sejnowski, 1995; Agnati and Fuxe, 2000; Diamond, 2002; Agnati et al., 2008) and provides an ideal form of communication between neighboring neurons as has been observed in other thalamic areas (Bright and Brickley, 2008; Errington et al., 2011; Bright and Smart, 2013; Herd et al., 2013; Ye et al., 2013).

#### MT Outputs

MT projects to prefrontal cortex (Middleton and Strick, 1994), motor cortex (Hoover and Strick, 1999), supplementary motor area (SMA) and pre-SMA (Akkal et al., 2007). Axonal processes from ventromedial (VM) and ventral anterior (VA) thalamic nuclei terminate in L1 (Donoghue and Ebner, 1981; Arbuthnott et al., 1990; Desbois and Villanueva, 2001; Mitchell and Cauller, 2001; Kuramoto et al., 2009, 2015; Rubio-Garrido et al., 2009).

**Figure 2:**
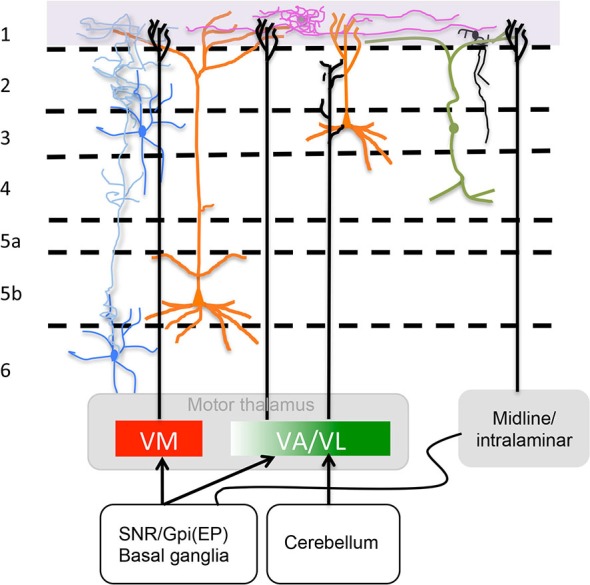
**Motor thalamus (MT) and related midline/intralaminar thalamic connections to layer 1.** Depicted in the rectangles at the bottom are projections from MT divided according to a direct VM and rostromedial VA projection to L1 and a caudolateral VA/VL projection that does not reach L1 as described by Kuramoto et al. (2015) (see “MT Inputs” Section ) illustrated also is the minor input from midline/intralaminar nuclei to L1 via the basal ganglia (see “Midline/Intralaminar Nuclei” Section). Specific L1 neurons are depicted neurogliaform (purple) and single bouquet (black) (see “Layer 1: The Crowning Enigma” Section). The rectangle at the top marks where axons from specific neurotransmitter producing nuclei (i.e., ACh, NE, 5HT and DA) run along L1. Sketched are also important neuronal processes mingled in L1 from axons of Martinotti cells (blue), vertical dendrites from bipolar interneurons that run horizontally once in layer 1 (green) and apical dendritic tufts of layers 2/3 and 5 pyramidal cells (orange) (see “Neuronal Processes that Mingle in Layer 1” Section). For accurate afferent arborizations consult Arbuthnott et al. (1990), Kuramoto et al. (2009) and Cruikshank et al. (2012).

VM neurons project to extensive motor associated cortical areas including the forelimb and hindlimb regions (Tennant et al., 2011; Deffeyes et al., 2015). These results are consistent with the findings of Arbuthnott et al. (1990) following antidromically driven VM neurons over a similarly extensive cortical area. The other areas that receive fibers from VM according to Kuramoto et al. (2009) are primary somatosensory (S1) and associated sensory orbital and cingulate areas. Figure 2 presents a sketch of afferents to L1, its neuronal types some of neuronal processes found at this level.

#### MT Functional Output

The motor function of MT reflects the function of its afferent nuclei: optimization of motor sequences, sensory motor control, switching of attention and decision-making of cerebellum e.g., D’Angelo et al. (2011) and attention, implicit learning, habit formation and selection of appropriate motor activity of basal ganglia e.g., Lanciego and Vázquez (2012). How this information is consolidated and expressed depends not only on integrative processes in MT but also on the reentrant cortical input from thalamus (Magill et al., 2004; Bosch-Bouju et al., 2013; Nakamura et al., 2014) and the anatomical reality of a reentrant thalamic pathway to L1.

Initial functional evidence of MT (VA, VM) in relation to basal ganglia output indicated that increases in GPi(EP) and SNR activity resulted in decreased VM activity (Deniau et al., 1978; Patino and Garcia-Munoz, 1985) and increased cortical activity (Tanibuchi et al., 2009). Monosynaptic inhibitory postsynaptic potentials were recorded in VM to stimulation of SNR or GPi(EP) (MacLeod et al., 1980; Ueki, 1983). Recently it has been reported that the similar electrophysiological characteristics of VA and VM suggest they could form a single nucleus recipient of inputs from basal ganglia (Nakamura et al., 2014). Coherence between cortical oscillatory activity (electrocorticograms, ECoG) and action potentials has been reported for cortex-basal ganglia (Magill et al., 2004) or cortex-thalamus (Nakamura et al., 2014). Nakamura et al. (2014) observed that MT neuronal spike discharges are phase-locked to ongoing cortical slow oscillations, and that the two neuronal populations of MT (defined by their GLU or GABA inputs) preferentially discharge at the ascending phase of the cortical oscillation.

VM and VA are assumed to carry information about movement from basal ganglia, though it will be strongly modified information. Deep brain stimulation in the subthalamic nucleus changes dramatically MT responses, decreases beta oscillations and improves Parkinson’s disease symptoms (Anderson et al., 2015) possibly via cortex. Moreover, thalamic deep brain stimulation is not only an effective treatment for movement disorders but also for pain and epilepsy. Nociceptive neurons are located in the lateral parts of VM (lateral to MT) they respond to painful stimulation of the whole body in rats and project to the entire layer 1 of the dorsolateral neocortex (Monconduit and Villanueva, 2005) and cortical epileptic activity during absence seizures is accompanied by rhythmic burst activity in VM (Paz et al., 2007), although the terminals seem widely spread in cortex L1 inhibitory neurons could target specific pyramidal cells.

### Midline/Intralaminar Nuclei

The lateral, ventral and posterior groups of midline and intralaminar nuclei interact with MT and its afferent sites according to Van Der Werf et al. (2002). The ascribed functions of these three groups are cognitive for the lateral (centrolateral, CL; centromedian anterior, CM; and paracentral, PC nuclei), sensory for the ventral (rhomboid, Rh; reuniens, Re; centromedian, CM and posterior, Po) and multisensory for the posterior group (parafascicular, Pf). Among the midline/intralaminar thalamic nuclei Rh, Re and Pf are consistently reported to project to striatum and also to cortical L1 (Berendse and Groenewegen, 1990; Mohammed and Jain, 2014). Optical stimulation of midline thalamic neurons (e.g., Re, PC, CM and Rh) preferentially drives L1 interneurons that often trigger feedforward inhibition of other L1 interneurons and L2/3 pyramidal neurons in medial prefrontal and secondary motor cortex (Cruikshank et al., 2012).

Projections from midline/intralaminar thalamus on their way to cortex on occasions bifurcate to reenter basal ganglia (Killackey and Ebner, 1973; Cesaro et al., 1979; Jinnai and Matsuda, 1981; Royce, 1983; Macchi et al., 1984) and in other cases separate groups of neurons send axons to striatal and cortical targets (Sadikot et al., 1992). Figure 2 illustrates ascending inputs to L1 (motor areas) from MT and midline/ intralaminar nuclei. The topic of dual projections is discussed by Jones and Leavitt (1974). Afferents from these nuclei to striatum have been reported in *monkeys* (Macchi et al., 1984; Nakano et al., 1990; Fenelon et al., 1991; Sadikot et al., 1992; Gimenéz-Amaya et al., 1995) *cats* (Sato et al., 1979; Beckstead, 1984; Takada et al., 1986) and *rodents* (Dube et al., 1988).

Gimenéz-Amaya et al. (1995) raised important questions regarding the double thalamic projection to striatum and cortex: Is their function modulatory at both ends? Are the contacted neurons a subset of neurons with common outputs or functions? Axonal branches from thalamic afferents that synapse in striatum on their way to cortex may contribute to striatal multisensory responses (Reig and Silberberg, 2014). Since the early work it was suggested that some afferents on L1 originated from the midline/intralaminar nuclei (Jones and Powell, 1969, 1970a,b; Strick, 1973; Strick and Sterling, 1974). Medium to large caliber axons from VA afferents converged with fine caliber afferents from intralaminar nuclei in L1 (Killackey and Ebner, 1973). Concurrent afferent information from MT and intralaminar nuclei to L1 and striatum may contribute to the animal’s awareness of position in space necessary for posture and head orientation (Barter et al., 2015) and also contribute to improvement of symptoms of Parkinson’s disease induced by deep brain stimulation (Jouve et al., 2010).

## Responses Mediated by Activation of Layer 1

The evidence presented in this section is a summary of the neuronal circuitry activated by L1 or compilation of experimental observations from different cortical areas i.e., S1, medial prefrontal, neocortical and of course specific motor cortex responses relevant to this review. Although the summary underlies general features, important anatomical distinctions between motor/frontal cortex and other cortical areas must be considered (Weiler et al., 2008).

### Activation of the Distal Tuft of Pyramidal Cells in L1

Electrophysiological responses of L1 interneurons are mediated by excitatory GLU (i.e., AMPA, kainite and NMDA) and inhibitory GABA_A_ receptors (Li et al., 2012). Thalamic afferents are in intimate contact with the distal part of the apical dendrite or dendritic tuft that reach L1 and provide a substantial AMPA and NMDA-mediated excitatory synaptic drive that generates subthreshold and suprathreshold voltage responses through the tuft. AMPA receptors mediate unitary depolarizing potentials and NMDA receptors mediate an extensive depolarizing input that leads to the generation in the dendritic tuft of calcium-dependent regenerative action potentials. In summary, inputs to L1 produce regenerative calcium spikes that can induce sodium axosomatic action potentials (Larkum and Zhu, 2002; Larkum et al., 2009). A general organization of cortical synaptic interactions emphasizes a descending influence on cortical output from layer 2/3 to layer 5 for rat S1 (Jiang et al., 2013) as well as motor cortex (Weiler et al., 2008) previously believed to have a predominantly horizontal synaptic interaction.

### Influence of the Distal Dendritic Tuft in L1 on Cortical Output

The presence of a cooperative integration between the distal dendritic tuft and the axosomatic compartment of pyramidal cells has been studied following co-activation of both compartments. It has been observed for example, that axosomatic action potentials can be generated by the coincidence of back propagation of action potentials and the effect of distal dendritic excitatory potentials (Larkum et al., 2004) and that volleys of excitatory postsynaptic potentials generated from distal apical dendritic sites facilitate action potential discharges (Williams, 2005).

Expression of specific channels in dendritic tufts that mediate different conductances such as the voltage-activated potassium outward conductance (K_v_ channels) or the hyperpolarization-activated current (*I*_h_) plus the effects of specific activation of neurotransmitter receptors are important mechanisms for microcircuit control. K_v_ channels in the distal apical dendrite of layer 5 pyramidal neurons control the interaction between subthreshold tuft excitatory input and trunk spike generation (Harnett et al., 2013). These channels are co-localized with hyperpolarization activated cyclic nucleotide-gated nonselective cation (HCN) channels that are expressed at a high density in the tuft (≈85 channels/μm^2^) and at low density in the somatic region with two different effects: an inhibitory action in the tuft that controls initiation and propagation of dendritic spikes, and an excitatory action in the soma that decreases the threshold for action potentials (Harnett et al., 2015). Also *I*_h_-mediated currents make corticospinal neurons susceptible to attenuation of GLU responses but not corticostriatal neurons. Similarly, the blockade of *I*_h_ results in increased layer 2/3-driven spiking in corticospinal, but not in corticostriatal neurons (Sheets et al., 2011). This emphasizes differential influences of L1 on cortical output.

#### Modulatory Role of Neurotrasmitters Released in L1

The neuromodulatory role of axons from several neurotransmitter systems (e.g., DA, NE, 5HT, ACh) on L1 is also important in the control of tuft currents and local interneurons. For example, α-adrenergic agents decrease *I*_h_ in corticospinal neurons thereby amplifying the impact of excitatory postsynaptic potentials with an increase in action potentials (Sheets et al., 2011). Likewise, the synergistic action of DA D1 and D2 receptors induces a depolarizing shift in the *I*_h_ activation curve that results in depolarization of L1 interneurons that can alter tonic cortical inhibition (Wu and Hablitz, 2005; Zhou et al., 2013).

Other neurotransmitters acting on L1 are also relevant for cortical function. For example, ascending ACh axons extend preferential terminations in L1 (Kristt et al., 1985) where neurons express high concentrations of nicotinic receptors (Komal et al., 2015), and an inhibitory interaction of DA D1/5 receptor on α7 nicotinic receptors has been observed in prefrontal L1 (Komal et al., 2015). This DA-Ach interaction can have important consequences in learning and memory. Optical stimulation of cholinergic axons induces excitatory potentials in L1 neurons and generates di-synaptic nicotine receptor-induced prolonged postsynaptic inhibition in layer 2/3 that could be associated with the effects of nicotine on arousal (Arroyo et al., 2012).

### Activation of Layer 1 in Motor Cortex

L1 at the level of motor cortex contains afferents from MT specifically VM and VA (Herkenham, 1979; Arbuthnott et al., 1990; Kuramoto et al., 2009, 2015; Rubio-Garrido et al., 2009) and from midline/intralaminar nuclei (e.g., Re, PC, CM, Pf and Rh; Berendse and Groenewegen, 1990; Cruikshank et al., 2012). It seems that stimulation of these afferents in L1 can produce changes in synaptic efficacy in motor cortex, for a review see Yu and Zuo (2011). Reorganization of motor representations is a common phenomenon seen after peripheral transection of nerves, repetitive limb movement or motor learning results (Elbert et al., 1995; Sanes and Donoghue, 2000; Harms et al., 2008; Plowman and Kleim, 2010).

#### Long-term Changes in Dendritic Spines of Pyramidal Neurons are Facilitated by L1 Stimulation

Direct and indirect activation of tufts of apical dendrites by brief stimulation of L1 *in vitro* induces long-term depression of layer 2/3 neurons (Walcott and Langdon, 2001). These changes in postsynaptic response are associated to structural changes likely related to activation of immediate early genes for instance, the activity-regulated cytoskeleton-associated proteins (Arc) and cFos proteins are increased in layer 2/3 and 180 μm from pia in motor cortex as a consequence of learning complex motor tasks (Kleim et al., 1996; Cao et al., 2015). Structural changes include increases in dendritic spine density and cortical map expansion of limb or paw representation. Increases in synapse/neuron ratio are reported for motor cortex layers 5 and 2/3 contralateral to the trained forelimb (Kleim et al., 1996) likely related to consolidation of learning (Kleim et al., 2004). Enlargement of dendritic spines in L1 (Harms et al., 2008) are also a good indicator of modifications of synaptic connectivity and the influence of this layer on the apical tuft of pyramidal neurons.

Recent *in vivo* visualization of dendritic spines has revealed specific neuronal changes related to motor activity and learned tasks in layer 2/3 of motor cortex in mice under head fixation. Ensembles of neurons display calcium transients in layer 2/3 during performance of a learned task (Komiyama et al., 2010; Huber et al., 2012; Hira et al., 2013; Masamizu et al., 2014) and a parallel pruning and growth of dendritic spines likely important for task performance occurs in the apical tuft of neurons active during acquisition of a task in layer 5 (Xu et al., 2009; Yang et al., 2009) and layer 2/3 (Peters et al., 2014; Ma et al., 2015). The presence of DA is crucial for dendritic spine turnover of layer 5 pyramidal tuft (Guo et al., 2015).

#### Neurotransmitters Released in L1 Modulate Plasticity in Motor Cortex

Learning changes information processing in cortical microcircuits; interest on how neuronal interactions between cortical layers ultimately result in behavior is of outmost relevance. Letzkus et al. (2011) have studied how Ach released in L1 following aversive foot shocks modifies cortical microcircuits involved in learning. Ach produces a nicotinic-dependent excitation of L1 neurons that results in inhibition of layer 2/3 palvalbumin interneurons. This inhibition in turn disinhibits pyramidal neurons. In order for mice to associate a sound with foot shock and express fear, the disinhibition of pyramidal neurons associated with foot shock must take place; pharmacological or optical blockade of pyramidal neuron disinhibition prevents learning. Most likely, similar effects induced by other neurotransmitters released in L1 (e.g., DA and 5HT) are involved in microcircuits involved in learning.

#### Horizontal L1 Connections between Motor and Sensorimotor Cortex

L1 forms cortico-cortical connections that provide feedback during behavioral tasks. Whisker touch activates L1 of vibrissal motor cortex that in turn sends excitatory input to L1 of S1 barrel cortex to excite dendritic tufts of layer 5 pyramidal neurons (Xu et al., 2012). The large calcium signals recorded in L1 in the S1 cortex during active facial whisker-object contact recorded by these authors in another set of experiments reinforce the concept of a top-down processing of behavioral relevant outputs as well as the horizontal cortico-cortical influence (Harnett et al., 2013).

## Summary and Conclusions

### Highlights on Advances in the Field

- Motor-related information from basal ganglia and cerebellum enters motor cortex via MT. This thalamic region has recently received a different demarcation thanks to new anatomical methods with very good resolution. As a result VA/VL has been divided in rostromedial and caudolateral areas immunoreactive to GAD67 and VGluT2, respectively. VM and rostromedial VA/VL receive GABAergic input from basal ganglia (SNR and GPi(EP)) and caudolateral VA/VL receives GLU terminals from cerebellum. VM and rostromedial VA/VL project to L1 and caudolateral VA/VL to layer 2/3.
- Apart from cerebellar and basal ganglia influences relayed to motor cortex via MT, indirect cerebellar inputs reach basal ganglia through midline/intralaminar outputs.
- Plastic related changes in spines of pyramidal dendritic tufts make learning-related structural changes a likely substrate for learning in L1.

### Voids in the Field that will be Interesting to Fill

- What is the role of the large synaptic terminals from SNR to VM described as “detonator drives” that typically favor neurotransmitter spillover and volume transmission?
- What is the function of the synaptic contacts made in basal ganglia from MT and midline/intralaminar axons as they travel to motor cortex? Which neurons are being contacted?
- What is the function of the electrically coupled elongated neurogliaform interneurons?
- Is 5HT released in L1 involved in learning-induced dendritic changes as are other neurotransmitters released in the area?

### Speculations on the Function of Layer 1

The excitatory input of L1 as studied in many cortical areas regulates the active regenerative electrical activity of dendrites of pyramidal cells of layer 2/3 and 5. This excitatory top down control on the dendritic tuft is crucial for integration and further generation of axosomatic discharge.

Recent combined electrophysiological and behavioral observations indicate that L1 can be considered as a driver of pyramidal neurons with important behavioral consequences such as attention, expectation, anticipation, and action command.

We would like to consider projections from MT and midline/intralaminar nuclei to L1 as a regulatory entity of pyramidal cell excitability in motor cortex. These projections can provide the necessary binding input to trigger or suppress final patterns of activity that would ultimately generate appropriate behavioral responses. Motor thalamus has already been labeled a “super integrator” (Bosch-Bouju et al., 2013) and the MT and midline/intralaminar nuclei projections to L1 provide a step further in the integration of motor, cognitive and motivational aspects to produce, in collaboration with thalamic inputs that ultimately reach layer 5, an appropriate response according to the surrounding environment. Hence, projections from MT and midline/intalaminar nuclei could resolve conflicting alternative response patterns and give continuity to cortical function as proposed by Edelman and Gally (2013) and Damasio (1989).

A source of concern when dealing with MT and midline/intralaminar afferents to L1 is the extent of their projection as observed in other neocortical areas. It is hard to conceive the point-to-point modulation suggested by some electrophysiological results e.g., Jiang et al. (2013). Perhaps there are two kinds of inputs, one that involves a restricted command and another that provides a wide-range informative action as suggested by anatomical studies e.g., Arbuthnott et al. (1990). A mundane example could be a “specific” reverse 911-emergency phone call ordering individual immediate evacuation vs. a “general” regional sound alarm of an approaching hurricane. Does L1 command both types of system? There is still plenty of mystery in the superficial cortical layer but at least forefront tools are shedding new light and making the future an exciting time to be studying this mysterious crown of the cortex.

Changes in dendritic spines induced by motor activity and learning observed in dendritic tufts in L1 underline the functional rewiring observed as changes in spine morphology and neuronal dynamics (Yuste and Bonhoeffer, 2001; Holtmaat and Svoboda, 2009).

## Conflict of Interest Statement

The authors declare that the research was conducted in the absence of any commercial or financial relationships that could be construed as a potential conflict of interest.
